# Navigating the genomic instability mine field of osteosarcoma to better understand implications of non-coding RNAs

**DOI:** 10.32604/biocell.2022.020141

**Published:** 2022-06-13

**Authors:** Kaniz FATEMA, Zachary LARSON, Jared BARROTT

**Affiliations:** Biomedical and Pharmaceutical Science, Idaho State University, Pocatello, 83209, USA

**Keywords:** Osteosarcoma, Epigenetics, Long non-coding RNA, Circular RNA, MicroRNA

## Abstract

Osteosarcoma is one of the most genomically complex cancers and as result, it has been difficult to assign genomic aberrations that contribute to disease progression and patient outcome consistently across samples. One potential source for correlating osteosarcoma and genomic biomarkers is within the non-coding regions of RNA that are differentially expressed. However, it is unsurprising that a cancer classification that is fraught with genomic instability is likely to have numerous studies correlating non-coding RNA expression and function have been published on the subject. This review undertakes the formidable task of evaluating the published literature of noncoding RNAs in osteosarcoma. This is not the first review on this topic and will certainly not be the last. The review is organized with an introduction into osteosarcoma and the epigenetic control of gene expression before reviewing the molecular function and expression of long non-coding RNAs, circular RNAs, and short non-coding RNAs such as microRNAs, piwi RNAs, and short-interfering RNAs. The review concludes with a review of the literature and how the biology of non-coding RNAs can be used therapeutically to treat cancers, especially osteosarcoma. We conclude that non-coding RNA expression and function in osteosarcoma is equally complex to understanding the expression differences and function of coding RNA and proteins; however, with the added lens of both coding and non-coding genomic sequence, researchers can begin to identify the patterns that consistently associate with aggressive osteosarcoma.

## Introduction

Osteosarcoma is the most common form of bone cancer and is the third most common cancer among adolescents. It is an aggressive cancer that frequently metastasizes within a year of forming (Faisham et al., 2017; Herzog, 2005; Mirabello et al., 2009; Tang et al., 2008). Over 40 years ago, long-term, disease-free survival of patients with high-grade osteosarcoma radically improved from less than 20% to greater than 60% with the advent of combinatorial, cytotoxic chemotherapy (Link et al., 1986). The most common cocktail of adjuvant chemotherapy to treat high-grade osteosarcoma consists of cisplatin, methotrexate, and doxorubicin (Carrle and Bielack, 2006). Despite the herculean reversal in dismal outcomes 40 years ago, there remains an enigmatic fraction of patients who fail to exhibit a durable response. Efforts have been made to genomically identify responders vs. non-responders in attempts to guide non-responders early to other therapeutic alternatives. However, due to the high level of genomic complexity, this has largely been unproductive. Osteosarcoma is the quintessential example of genomic instability, with numerous point mutations, INDELS, and structural variants throughout the entire genomic landscape. Most of the identified genomic variations are seen as inconsequential; although, the non-coding variants might hold the key to unlocking the mystery underlying the differences between survivors and non-survivors. Herein this review, the most common epigenetic mechanisms that can contribute to chemosensitivity are discussed; including numerous mechanisms that involve non-coding RNAs.

## Epigenetic Control of Gene Expression

Precision medicine is centralized around the dogma of molecular biology. This states that DNA is the fundamental coding material that transcribes sequences into RNA, and RNA is translated into protein resulting in phenotypic cellular behavior (Crick, 1970). Thus, changes in any step along the process can impact how cells act. While DNA mutations can alter this pattern, factors outside of DNA mutations can equally impact gene expression levels to change cell behavior. This is referred to as epigenetic control (Baylin and Jones, 2016). Although DNA methylation and histone modifications are the most commonly studied and well-understood mechanisms of epigenetic control over gene expression, another new angle that has been under investigation in recent times is regulation by non-coding RNAs (Yang et al., 2020). With progress made in personalized medicine and epigenetic therapeutics in cancer, harnessing this unique interplay between non-coding RNAs and the other epigenetic enzymes might be an excellent alternative to address the therapeutic challenges in osteosarcoma.

### Non-coding RNAs

The entire mammalian genome can be transcribed to some level, yet there remain thousands of RNA transcripts that do not code for proteins. These are known as non-coding RNAs (ncRNAs) (Palazzo and Lee, 2015; Yan and Bu, 2021; Yang et al., 2020; van Bakel et al., 2010). Their expression level and the function are very controversial. Previously known as non-functional, ‘noise’ or ‘junk’ of transcriptome, according to recent reports, much of those transcriptions are likely functional (Richard Boland, 2017) and associated with disease pathogenesis and progression, especially in cancers and neurodegenerative disorders. They can alter gene expression at pre-transcription, transcription, and posttranscription levels and regulate numerous cellular processes related to cancer initiation and progression (Schmitt and Chang, 2016; Yang et al., 2020; Zhu et al., 2019). A growing body of evidence emphasize that ncRNA’s control over genes and chromosomal modifications are attributed to their interactions with the chromatin remodeling complexes, histone modifiers, or DNA methyltransferases (Chen and Xue, 2016; Costa, 2008; Peschansky and Wahlestedt, 2014; Ramassone et al., 2018; Yu, 2009). Differential expression and stability of ncRNAs, especially in blood or urine hold great diagnostic and prognostic biomarker potential in various cancer types including osteosarcoma (di Fiore et al., 2013; Wu et al., 2019). Indeed, some ncRNAs have already been proposed to be the circulating biomarkers owing to their correlation with osteosarcoma progression and metastasis, clinical stage, and patient outcome (Botti et al, 2019).

Based on their size, shape and genomic location, ncRNAs are divided into three major classes, long ncRNAs (lncRNAs), circular RNAs (circRNAs) and short ncRNAs (micro RNAs, short interfering RNAs, piwi RNAs) (Palazzo and Lee, 2015; Wu et al., 2019; Yan and Bu, 2021). Long ncRNAs (lncRNA) are the linear gene transcripts with limited protein-coding capacities, over 200 NT long and regulate gene expression at pre-transcriptional, transcriptional, and post-transcriptional levels (Yan and Bu, 2021). Depending on their expression pattern and biological function, lncRNAs can be classified as sense, antisense, bidirectional, intron, intergenic, or enhancer-lncRNAs (Al-Rugeebah et al., 2019; Palazzo and Lee, 2015). Circular RNAs (circRNA) are another long transcript type but unlike lncRNAs, these single-stranded RNAs form a covalently closed continuous loop (Chen and Huang, 2018). Regulation of gene expression by both lncRNAs and circRNAs includes miRNA decoy/sponging, therefore interacting with DNA, RNA, and proteins (Zhang et al., 2020c). MicroRNAs (miRNA) are the small, 19 to 22 nucleotide base pair sequences, they can inhibit translation or result in the degradation of target mRNA by forming RNA-induced silencing protein complex (RISC) (Llobat and Gourbault, 2021). PIWI-interacting RNAs (piRNA) are the 24 to 32 long transcripts mainly expressed in the germline, derived from single-stranded RNA, and processed by Dicer-independent process. piRNAs are very well known for their function in repressing transposable elements and epigenetic regulation of gene expression (Costa, 2008; Zeng et al., 2020).

### Non-coding RNAs and genomic instability

DNA damage response, mediated by a well-constructed regulatory network, is a vital part of maintaining genomic integrity. Decades of research in ncRNAs led to a significant bidirectional regulatory loop between the differential expression of ncRNAs and regulation of DDR-associated genes expression (Malakoti et al., 2021; Zhang and Peng, 2015). ncRNAs, especially lncRNAs, miRNAs and circRNAs, have been found to play multifaceted roles in DDR such as acting as DDR sensor or transducer; thereby, repairing DNA, causing cell cycle arrest, or inducing apoptosis (Khanduja et al., 2016; Malakoti et al., 2021; Zhang and Peng, 2015). It is not surprising that many of these pathways are also interlinked with chromatin remodeling or histone modifications. However, even with most extensive research and understanding of ncRNAs and DDR pathways, it remains questionable how ncRNAs and DDR pathways align together in maintaining cellular integrity. Thus, in this review, we sought to understand whether there is any correlation between epigenetic regulation of the ncRNAs with one of the major hallmarks of cancer: genomic instability.

## Long Non-Coding RNAs

lncRNAs can directly or indirectly affect almost all of the hallmarks of cancer (Hanahan and Weinberg, 2011; Yan and Bu, 2021) and their oncogenic or tumor-suppressive role can be regulated genetically and/or epigenetically (Zhang et al., 2020c). Since discovery of the regulatory mechanisms of the earliest lncRNAs (Lo et al., 2014), many functional lncRNAs were found to work through epigenetic mechanisms, such as, H19 (imprinted maternally expressed transcript), Xist (X-inactive-specific transcript), HOTAIR (Hox transcript antisense intergenic RNA), etc. (Chan et al., 2014; Clemson et al., 1996; Fazi et al., 2018; Zhang et al., 2015; Zhang et al., 2020c; Zhao et al., 2008).

The expression pattern of lncRNAs and their function in tumorigenesis is well investigated in osteosarcoma (Table 1). A recent transcriptome profiling study based on the TARGET data detected a total of 13,903 expressed lncRNAs and their integrative gene expressions and SCNA analysis revealed 167 novel driver lncRNAs (including 2 previously reported lncRNA PVT1 and ZFAS1) to be associated with osteosarcoma (Luo et al., 2019). Another microarray analysis noted 25,733 expressed lncRNAs in human osteosarcoma, among which 403 were upregulated and 798 were downregulated when comparing osteosarcoma tissues to adjacent normal tissues (Li et al., 2018). Some lncRNAs were also highlighted as circulating biomarkers due to their stability, significantly higher expression in body fluids (especially in serum/plasma of osteosarcoma patients) and their correlation to disease stage or metastatic potential; for example, lncRNA TUG1, UCA1, HNF1A-AS1, MALAT-1, FAL-1 and ATB, etc. (Botti et al., 2019). All of these data imply that many lncRNAs are involved in osteosarcoma occurrence, chemoresistance and metastasis or relapse which could be exploited as potential diagnostic, prognostic biomarkers, and therapeutic targets.

**FIGURE 1.**
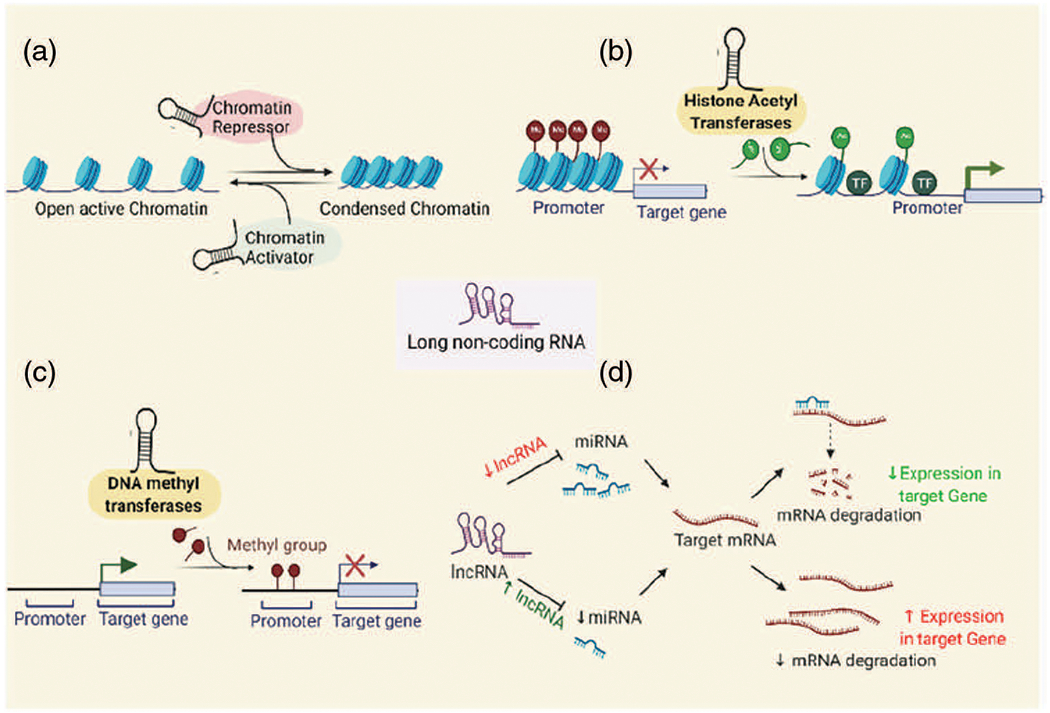
Epigenetic modifications of the lncRNAs to regulate gene expression: (a) via chromatin modification and remodeling by interacting with the epigenetic activator or repressive complex members, (b) histone modifications to alter chromatin structure, (c) via Promoter DNA methylation, (d) via ceRNA networking (miRNA sponging).

Until recently, several differentially expressed lncRNAs have been identified in different cancers owing to their association and interaction through epigenetic modifications such as histone modification and/or chromatin remodeling (e.g., lncRNA XIST, MALAT1, HOTAIR, ANRIL, HULC, GCLnc1, FENDRR, UCA1, TCF7, GAS5, NEAT1, PVT1); DNA methylation (e.g., lncRNA H19, DACOR1, PTENP1); and CpG Island methylation at the Imprinting Control Regions (e.g., lncRNA TP53TG1, MEG3), competing endogenous RNA (ceRNA) networks (e.g., lncRNA CASC2/miR-183/Sprouty2; NKAPP1-miR-21-5p-PRDM11, MSTO2P-miR-29c-3p-EZH2 and RPLP0P2-miR-29c-3p-EZH2) (Gao et al, 2019; Lou et al, 2020; Wei et al, 2017). lncRNAs are generally found to carry out their epigenetic modifications via-(a) chromatin modification and remodeling, (b) histone modification and nuclear body localization, (c) altering chromosome structures by interacting with the SWI/SNF complex, (d) inducing DNA methylation and/or (e) through interactions with micro RNAs by acting as miRNA sponges or via ceRNA (competitive endogenous RNA) networking (Shin et al, 2020; Zhang et al, 2020c) (Fig. 1).

### Chromatin modifications and DNA methylation patterns

Around 25% of all the intergenic lncRNAs have been found to interact with the chromatin-modifying proteins especially via interacting with Histone modifying enzymes (e.g., histone acetyl transferases, histone deacetylases) and/or incorporating PRC2 complex members (e.g., EED, SUZ12 and EZH2), and facilitate transcriptional and post-transcriptional regulation of target genes (Cantile et al., 2020; Ren et al., 2019a). EZH2 (Enhancer of Zeste Homolog 2), which is a histone methyltransferase, a critical element of the multiplex-suppression complex called Polycomb Repressive Complex 2 (PRC2). EZH2 functions through the trimethylation of lysine in histone H3 and its aberrant expression has been heavily investigated in cancers (Czermin et al., 2002; Yang et al., 2018). Several studies have documented the involvement of EZH2 with lncRNAs in osteosarcoma. In addition to these, the alteration in the methylation patterns (by binding DNMTs, e.g., DNMT1, DNMT3A, and DNMT3B) mediated by lncRNAs have also been associated with the pathogenesis and progression of osteosarcoma. Other mechanisms involve ubiquitination or phosphorylation in important onco- or tumor suppressor genes, and a strong regulatory network amongst the lncRNAs and miRNAs could also be exploited given their implicit regulation of genes expression. The following sections list some of the most significant lncRNAs in osteosarcoma that have been implicated for their biomarker potential.

*lncRNA HOTAIR* is one of the broadly studied ncRNAs HOTAIR is an important EMT regulator and has been implicated in the pathogenesis of several cancers. HOTAIR inhibits HOXD transcription through PRC2 recruitment, forming a heterochromatin and transcriptional gene suppression via H3K9 trimethylation (Zhang et al., 2015). In osteosarcoma, HOTAIR was found to positively regulate the global DNA methylation level and specifically DNMT1 expression, making it an interesting diagnostic marker and therapeutic target (Li et al., 2017b). ***lncRNA MALAT1*** (Metastasis Associated Lung Adenocarcinoma Transcript 1) located at chromosome 11q13.1, is suggested to be an oncogenic lncRNA in other cancers. Epigenetic regulation of MALAT1 in osteosarcoma has been investigated (Zhang et al., 2018b) especially with respect to the expression pattern of EZH2 (Li et al., 2017a). Zhang et al. (2018b) identified that MALAT1 regulates the expression of β-catenin and E-cadherin via the MALAT1/EZH2 axis which in turn changes the gene expression downstream of β-catenin. In another latest study, MALAT1 was also found to serve as a ceRNA network for HDAC4 (histone deacetylase 4), where it regulates osteosarcoma proliferation and apoptosis by upregulating HDAC4 via decoying miR-140-5p (Sun and Qin, 2018).

A majority of the antisense-lncRNAs influenced dysregulation by either methylation pattern or chromatin conformational changes, typically found to regulate expression of their opposite strand gene. For instance, ***lncRNA KCNQ1OT1*** (KCNQ1-opposite strand/antisense transcript-1) negatively regulates KCNQ1 gene via promoting DNMT1 expression in the KCNQ1 gene promoter region (Qi et al., 2019). lncRNA ***FOXD2-AS1*** (FOXD2 Adjacent Opposite Strand RNA 1) is robustly expressed in the osteosarcoma tissue specimens and cell lines (induced by transcription factor HIF-1α). FOXD2-AS1 was found to play a critical role in hypoxia-induced osteosarcoma tumorigenesis by interacting with the EZH2 and repressing p21 protein expression (Ren et al., 2019b; Zhang et al., 2021). The oncogenic ***lncRNA DANCR*** (Differentiation Antagonizing non-coding RNA) is overexpressed in many cancers and also promotes proliferation, migration and invasion in osteosarcoma (Pan et al., 2020). Several studies found the interaction between DANCR and EZH2 in many tumor types including osteosarcoma (Cheng et al., 2021; Wang et al., 2019; Zhang and Peng, 2017). When DANCR was knocked down, it lowered the EZH2 expression and activated both p21 and p27, hence inhibiting the osteosarcoma cell proliferation, migration, and invasion (Zhang and Peng, 2017). ***lncRNA HOXD-AS1*** epigenetically inhibits p57 by interacting with EZH2, thereby repressing the expression of p57 and aggravating osteosarcoma oncogenesis (Gu et al., 2018).

lncRNA interactions with EZH2 were seen again with ***FOXP4-AS1*** (forkhead box P4-AS1). Overexpression of FOXP4-AS1 was found to regulate osteosarcoma progression by downregulating LATS1 (large tumor suppressor 1) through binding LSD1 (lysine-specific demethylase 1) and EZH2 (Yang et al., 2018). The oncogenic ***lncRNA MIR100HG*** is another potential prognostic marker that promotes osteosarcoma progression via interacting with EZH2. MIR100HG epigenetically silences both LATS1 and LATS2 kinases by binding with EZH2 and thereby inactivating the Hippo signaling pathway (Su et al, 2019).

Hippo is an evolutionarily highly conserved pathway for the control of organ development and other cellular functions that are vital in oncogenesis (Calses et al., 2019; Harvey et al., 2013). The transcriptional activity of one of the downstream effectors of Hippo signaling, YAP (Yes-associated protein 1) was proved to be governed by ***lncRNA B4GALT1-AS1*** (Li et al., 2018) and ***XIST*** to maintain osteosarcoma tumor progression. Interestingly, YAP overexpression is also induced by aberrant Hedgehog signaling, which in turn causes overexpression of lncRNA H19 and is responsible for the pathogenesis of osteoblastic osteosarcoma (Chan et al., 2014). Recently, in a comprehensive study characterizing prognostic lncRNA, correlation analysis of copy number alteration (CNA) and lncRNA expression identified ***AC011442.1***, predicted to regulate AMPK and hedgehog signaling pathway thereby acting as an oncogenic driver in osteosarcoma (Gao et al., 2020).

Oncogenic ***lncRNA ZEB1-AS1*** (ZEB1 Antisense-1) has been implicated as a potential biomarker and therapeutic target due to its association with the opposite strand gene, ZEB1. ZEB1-AS1 can recruit histone acetyltransferase p300 to the promoter region of ZEB1 that results in an open chromatin structure and active transcription of ZEB1 promoting osteosarcoma proliferation and migration (Liu and Lin, 2016; Cheng et al., 2020). ***lncRNA HIF1α-AS1*** (hypoxia-inducible factor 1α-antisense-1) interacts with BRG1 (Brahma-related gene-1), this was suggested as a novel therapeutic agent for bone diseases as it was found to be an essential mediator of osteoblast differentiation regulated by acetylation (histone deacetylase sirtuin 1) (Xu et al., 2015).

### Crosstalk between lncRNAs and miRNAs

One of the ***MEG3*** (maternally expressed gene 3) gene transcripts, a 1.6 kb lncRNA situated in 14q32, is a very well-known tumor suppressor lncRNA in many cancer types (Li et al., 2013; Shen et al., 2019). The lost or reduced expression of MEG3 in different cancers has been associated with promoter hypermethylation and hypermethylation of the intergenic region (Al-Rugeebah et al., 2019; Modali et al., 2015; Sellers et al., 2019; Zhou et al., 2012). In osteosarcoma, MEG3 expression and function were mainly associated with ceRNA network or miRNA sponging mechanisms, e.g., sponging onco-miR664a, MEG3/miR-361-5p/FoxM1 axis, MEG3/miR-127/ZEB1 axis, etc. In most of the cases, MEG3 was suggested to be acting as a tumor suppressor and thereby a potential prognostic biomarker for osteosarcoma. Its overexpression was also able to prevent cell growth and metastasis by targeting oncogenes or by inhibiting signaling pathways like Notch and TGF-β (Shen et al., 2019; Sun et al., 2020; Zhang et al., 2017a). ***lncRNA CEBPA-AS1*** (CCAAT enhancer-binding protein alpha, aka LOC80054), that is usually downregulated in osteosarcoma (GSE16088) and other cancers (Ke et al., 2017), has recently been reported to inhibit osteosarcoma cell proliferation, differentiation, and enhance apoptosis by repressing the Notch signaling pathway via upregulating the expression of miR-10b-5p-mediated nuclear receptor corepressor 2 (NCOR2) (Xia et al., 2020). ***lncRNA-p21*** (also known as ***TRP53COR1***-tumor protein p53 pathway corepressor 1) has been reported to have *in vivo* and *in vitro* antitumor properties against wide range of tumors especially via cell cycle checkpoint regulation or regulating energy metabolism, or p53 and B-catenin pathway (Dimitrova et al., 2014; Wang et al., 2014; Yang et al., 2014; Yang et al., 2015; Yang et al., 2016). In osteosarcoma it was downregulated, and when overexpressed, it could upregulate the tumor suppressor PTEN (phosphatase and tensin homolog deleted on chromosome ten) level (Han and Liu, 2018). The tumor suppressor function of p-21 was via sponging miR-130b that significantly inhibited osteosarcoma proliferation (Han and Liu, 2018). The lncRNA SNHG10 (lncRNA small nucleolar RNA host gene 10) plays an important role in osteosarcoma growth via miR-182-5p sponging and the SNHG10/miR-182-5p/FZD3 axis maintain the activation of the Wnt/β-catenin signaling pathway (Zhu et al., 2020).

The oncogenic ***lncRNA AFAP1-AS1*** (actin fiber-associated protein 1 antisense RNA 1) has been proposed as a promising therapeutic target in osteosarcoma as it is found to be overexpressed and promoting the epithelial-mesenchymal transition of osteosarcoma through RhoC/ROCK1/p38MAPK/Twist1 signaling pathway (Shi et al., 2018). Recently, Fei et al. (2020) further strengthened this fact showing that AFAP1-AS1 promotes osteosarcoma progression by regulating the miR-497/IGF1R axis and targeting it could inhibit tumorigenesis both *in vitro* and *in vivo*. The ***lncRNA NBAT1*** (neuroblastoma-associated transcript 1), is recognized as a tumor suppressor lncRNA in some cancers. In osteosarcoma, the lower NBAT1 expression was associated with distant metastasis and poor prognosis, as it interacts with miR-21 and its downstream gene targets including PTEN, PDCD4, TPM1 and RECK (Yang et al., 2017). ***lncRNA PVT1*** (Plasmacytoma Variant Translocation 1) is a well-studied oncogenic lncRNA that was found to function as competing endogenous RNA or interact and stabilize the MYC protein (Sun et al., 2019; Tseng et al., 2014). Recently Chen et al. (2020) investigated the regulatory mechanism of PVT1 in osteosarcoma and identified that m6A demethylase ALKBH5-mediated demethylation of the PVT1 promotes osteosarcoma growth. The authors proposed PVT1 as a potential prognostic marker and ALKBH5-PVT1 to be a promising therapeutic target.

A novel interplay between ***lncRNA HOTAIR***, miR-126, and DNA methylation in osteosarcoma has been reported by Li et al. (2017b), where they found that HOTAIR can repress CDKN2A gene expression through DNA hypermethylation by suppressing miR-126 expression (a negative regulator of DNMT1). Therefore, the lncRNAHOTAIR-miR126-DNMT1-CDKN2A axis was proposed to be a novel therapeutic alternative, especially targeting HOTAIR due to its potential to increase osteosarcoma chemosensitivity toward DNMT1 inhibitors (Li et al., 2017b). ***lncRNA ANRIL*** (antisense non-coding RNA in the INK4 locus) is upregulated across many cancers. It is a 3.8 kB-long transcript expressed in the opposite direction from INK4A-ARF-INK4B which represses the expression of tumor suppressor gene p15 (INK4B) via recruiting PRC2 complexes (Kotake et al., 2011). The epigenetic crosstalk between ANRIL and the microRNAs was first documented in gastric cancer (Zhang et al., 2014a), its overexpression affects osteosarcoma cell proliferation, invasion, apoptosis (Cheng et al., 2017; Yu et al., 2018) and metastasis (Guan et al., 2018). Knockdown of ANRIL in the osteosarcoma cell lines was able to increase the Cisplatin-induced chemosensitivity via the upregulation of miR-125a-5p and reduction of its target gene STAT3 (Li et al., 2019). Consistent with other studies, this research indeed reveals the potential for targeting lncRNA-ANRIL/miR-125a-5p axis in the treatment of the chemoresistant osteosarcoma. The *lncRNA DANCR* has been documented to function by decoying miR-335-5p and miR-1972 in osteosarcoma and to facilitate ROCK1-mediated proliferation and metastasis (Pan et al., 2020). The major signaling pathways and ceRNA network that have been found to be associated with lncRNA *MALAT1* are the PI3K/AKT pathway, RhoA/ROCK signal transduction pathway, MALAT1/miR-509/Rac1 axis, MALAT1/miR-142-3p/miR-129-5p/HMGB1 axis (Cai et al., 2016; Dong et al., 2015; Liu et al., 2017a; Zhang et al., 2018a).

### Other epigenetic signatures

***lncRNA EPIC1*** (Epigenetically Induced lncRNA-1), despite playing an oncogenic role in other cancers through interacting with the oncogenic c-MYC protein (Wang et al., 2018), it has shown to have an opposite effect on osteosarcoma. It is able to inhibit the cell viability and invasion *in vitro* as well as suppress tumor growth in the osteosarcoma xenograft model by ubiquitin-mediated degradation of myocyte-specific enhancer factor 2, MEF2D (Zhao et al., 2019). ***lncRNA BLACAT1*** (Bladder cancer associated transcript 1) interacts with STAT3 and regulates the phosphorylation of STAT3 and contributes to the proliferation and migration of osteosarcoma cells (Dong and Wang, 2019).

## Circular RNAs

Another emerging new class of biomarkers for cancer development and progression are the circular RNAs (circRNAs). CircRNAs are the covalently closed/looped single-stranded non-coding RNA molecules, created by back-splicing of the pre-mRNA regulated by specific RNA-binding proteins (Bach et al., 2019; Zhang et al., 2020b). Previously they were considered as splicing errors, but recently many circRNAs are being discovered taking part in the post-transcriptional regulation of gene expression via different mechanisms.

**FIGURE 2.**
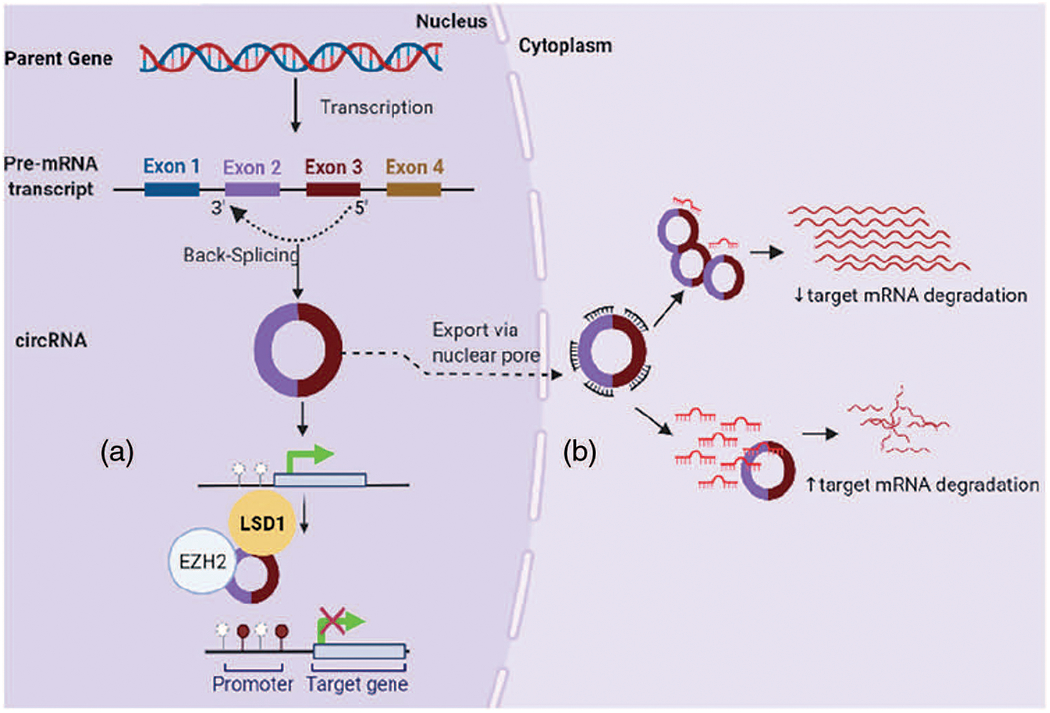
CircRNAs in osteosarcoma regulate gene expression: (a) by interfering with DNA methylation (e.g., binding EZH2), (b) via circRNA-miRNA-mRNA interaction.

In osteosarcoma, circRNAs modulate cell proliferation, migration and invasive properties mostly via circRNA-miRNA-mRNA interaction to regulate expression of specific onco- or tumor suppressor genes; for example, hsa_circ_0001564 (sponge miR-29c-3p), hsa-circ-0016347 (sponge miR-214), circGLI2 (sponge miR-125b-5p), circ-03955 (sponge miR-3662), circ-0001785 (sponge miR-1200), circPVT1 (circPVT1/miR-52b/FOXC2 axis), circ-NT5C2, hsa_circ_0092509, hsa_circ_0009910 (Chen and Huang, 2018; Fatema and Barrott, 2022; Jin et al., 2017; Liu et al., 2021; Wu et al., 2021; Wu et al., 2019). However, their function is not confined to sponging miRNA or proteins only. Accumulating evidence suggests a clear connection between the differential expression of circRNAs and the enzymes regulating DNA methylation or histone proteins (Bach et al., 2019; Chen and Huang, 2018; Jin et al., 2017; Lee et al., 2019) (Fig. 2).

A comprehensive characterization of circRNAs in around 1000 human cancer cell lines identified a strong association between the circRNAs and drug response, especially ***circMYC*** (associated with breast cancer cell proliferation) has shown a great sensitivity towards the drugs targeting histone acetylation (i.e., HDAC inhibitors Belinostat and Vorinostat) (Ruan et al., 2019).

Positive correlation between the histones and circRNAs has also been documented in several osteosarcoma cases (Table 2). For example, novel circRNA, ***circHIPK3*** (homeodomain interacting protein kinase-3) which has been found to promote HDAC4 expression via sponging of miR-637 to regulate osteosarcoma cell proliferation, migration, and invasion (Wen et al., 2021). However, earlier reports of circHIPK3 state that it has a tumor suppressor function and a clinical correlation where decreased expression associates with shorter overall survival (Long et al., 2018). Oncogenic ***circLRP6*** is highly expressed in osteosarcoma and its overexpression was associated with shorter patient survival (both disease-free and overall). Functionally, circ-LRP6 binds to both LSD1 and EZH2 and inhibits APC and KLF2 expression thereby promoting osteosarcoma progression (Zheng et al., 2019). In another study, Wu et al. (2019) focused on the underlying mechanism of ***circTADA2A*** which is abundantly expressed in osteosarcoma. TADA2A gene is part of the PCAF histone acetyltransferase complex and plays important role in chromatin remodeling, TP53 transcriptional activity, and regulating apoptosis via XRCC6 acetylation (Huang et al., 2012). This group concluded that circTADA2A targets an oncogene, CREB3 to promote osteosarcoma progression and metastasis via sponging to miR-203a-3p and emphasized circTADA2A-miR-203a-3p-CREB3 axis as a potent osteosarcoma-targeted therapy.

## Short Non-Coding RNAs–*Micro RNAs*

According to the miRBase v.22.1, the human genome encodes for approximately 2,654 mature microRNAs (Gregorova et al., 2021) and any single microRNA is capable of regulating the expression of hundreds of different genes (Kim et al., 2008; Plotnikova et al., 2019). Depending on their expression pattern and molecular targets in different cancer types, miRNAs can have either oncogenic or tumor suppressive function in tumorigenesis (di Leva et al., 2014). miRNAs can regulate gene expression via RNA interference (RNAi) mechanism, miRNA sponging mechanism (ceRNA network), other epigenetic processes such as interfering with DNA methylation, especially targeting DNA methyltransferases (Fabbri et al., 2007; Garzon et al., 2009; Llobat and Gourbault, 2021) as well as the histonemodifying complex members (Alvarez-Saavedra et al., 2011; Garzon et al., 2009).

### miRNAs and epigenetics

miRNAs can initiate transcriptional gene silencing or induce re-expression of methylation-silenced genes through restricting chromatin remodeling enzymes activity and/or altering DNA methylation status (Benetti et al., 2008; Fabbri et al., 2007; Wei et al., 2017; Yuan et al., 2011). Epigenetic mechanisms and chromosomal abnormalities have also been highlighted as the trigger to the aberrant expression of miRNAs in different cancers (di Leva and Croce, 2013; Vicentini et al., 2019). In 23 different types of tumors, 12% of all the miRNA genes associated with CpG islands were found inactivated by methylation (Gregorova et al., 2021). Moreover, the epigenetic modulators, such as histone methyltransferases, methyl CpG binding proteins, chromatin domain proteins, and histone deacetylases are also identified as potential targets of the miRNAs (Gregorova et al., 2021; Kim et al., 2008; Plotnikova et al., 2019). They have been found to modulate components of Polycomb complexes, e.g., targeting the EZH2 (miRNA-101, miR-26a), inhibiting stem cell factor BMI-1 (miR-128, miR-200c), promoting skeletal muscle differentiation (miR-214) (Peschansky and Wahlestedt, 2014). Differentially expressed histone deacetylases are also targets of miRNAs, but the conclusions often vary among tumor types (e.g., miR-449) (Buurman et al., 2012; Noonan et al., 2009).

### miRNA clusters and families in the cancer epigenetics

25% of all human miRNA genes are organized in clusters based on their genomic location (within <10 kB range) and expression profiles (kabekkodu et al., 2018). These clusters may contain the smallest to the highest number of miRNAs with similar biological function; whereby intergenic regions contain the most clusters compared to other locations. Two or more miRNAs with high sequence similarity are referred to as miRNA gene family and each family can be part of the same or different miRNA clusters depending on their function (Guo et al., 2014).

miRNAs within the same cluster regulate the expression of the onco-and tumor suppressor genes to promote carcinogenesis both genetically (e.g., SNPs) and epigenetically (e.g., CpG island hyper-and hypomethylation, etc.) (kabekkodu et al., 2018). Gregorova et al. (2021) highlighted the epigenetic mechanisms in different cancers that are found associated with the dysregulation of miRNA clusters or miRNA gene families such as-global or site-specific hypomethylation, CpG island promoter hypermethylation, and histone modifications, etc. According to that report, the most highlighted cancer associated miRNA clusters/families are-Let-7-5p/98-5p Family, miR-125-5p Family, miR-99-5p/100-5p Family; miR-34-5p/449-5p Family, miR-34b-5p/449c-5p Family; The miR-141-3p/200a-3p Family, miR-200ab-5p Family, miR-200bc-3p/429 Family, miR-200c-5p/550a-3p Family; miR-17~92a-1 Cluster, miR-106a~363 Cluster; miR-15-5p/16-5p/195-5p/424-5p/497-5p Family; miR-23-3p Family, miR-23b~24-1 Cluster, miR-23a~24-2 Cluster; miR-130-3p/301-3p/454-3p Family and miR-29-3p Family (Gregorova et al., 2021).

### miRNAs in osteosarcoma

Several miRNAs found deregulated in osteosarcoma compared to normal bone, osteoblasts, and mesenchymal stem cells, and some are also selectively expressed in osteosarcoma (Baumhoer et al., 2012; Jones et al., 2012; Llobat and Gourbault, 2021; Maire et al., 2011; Ramassone et al., 2018; Sarver et al., 2010; Thayanithy et al., 2012; Ziyan et al., 2011). miRNAs may well play both oncogenic and tumor suppressive roles depending on their target genes and pathways in osteosarcoma (Llobat and Gourbault, 2021). In a recent review on miRNAs in human osteosarcoma, Llobat and Gourbault (2021) compiled the miRNAs involved in osteosarcoma progression, in particular the clusters which have pivotal role in cancer hallmarks, for example the miR-17-92 and miR-106b-25 clusters. In a global microarray analysis of a panel of 19 human osteosarcoma cell lines, Naml0s et al. (2012) identified 177 differentially expressed miRNAs relative to normal bone, nearly half of which overlapped with two earlier studies, including the common miR-150. Among these, miR-126/miR-126*, miR-142-3p, miR-150, miR-223, miR-486-5p, and members of the miR-1/miR-133a, miR-144/miR-451, miR-195/miR-497 and miR-206/miR-133b clusters were found to be downregulated; miR-9/miR-9*, miR-21*, miR-31/miR-31*, miR-196a/miR-196b, miR-374a and members of the miR-29 and miR-130/301 families were found to be upregulated (Table 3).

All such differentially expressed miRNAs, miRNA clusters and families - having the similar biological function with the potential to regulate specific mRNAs of target genes-are promising diagnostic and prognostic markers for osteosarcoma (Lei et al., 2020; Ramassone et al., 2018; Zhang et al., 2015). A large number of miRNAs hold biomarker potential due to their differential expression in body fluids (especially in patients’ blood), their correlation with the response to chemotherapy and patient survival; for instance, ***miR-Let7A*** (Hua et al., 2018). Botti et al. (2019) recently in a review summarized the circulating miRNAs with biomarker potential in the diagnosis, prognosis, and clinical monitoring of osteosarcoma patients. According to them, ***miR-29*** family members (miR-29a, miR-29b, miR-29c), miR-199a-3p, miR-196a, miR-196b, miR-214, miR-574, miR-335, miR-9, miR-191, miR-221, miR-148, miR-195-5p, miR 320a, miR-421, miR-542, miR-95-3p, miR-21, miR-27a and miR-253-p are found highly expressed in patient serum and the miR-34 family members (miR-34a and miR-34b), miR-124, miR-205-5p, miR-133b, miR-206, miR-195, miR-152, miR-326, miR-145, miR-558, miR-497, miR-491 and miR-375 are significantly downregulated. Zhang et al. (2020a); Wu et al. (2011) proposed a novel diagnostic marker for predicting osteosarcoma metastasis, the plasma EV-packaged miR-101 (***EV-miR-101***), which the author indicated might serve as a useful circulating biomarker. Furthermore, the following sections will delineate the major markers of epigenetic control of miRNAs in osteosarcoma.

### DNA methylation and chromatin modifying miRNAs

**FIGURE 3.**
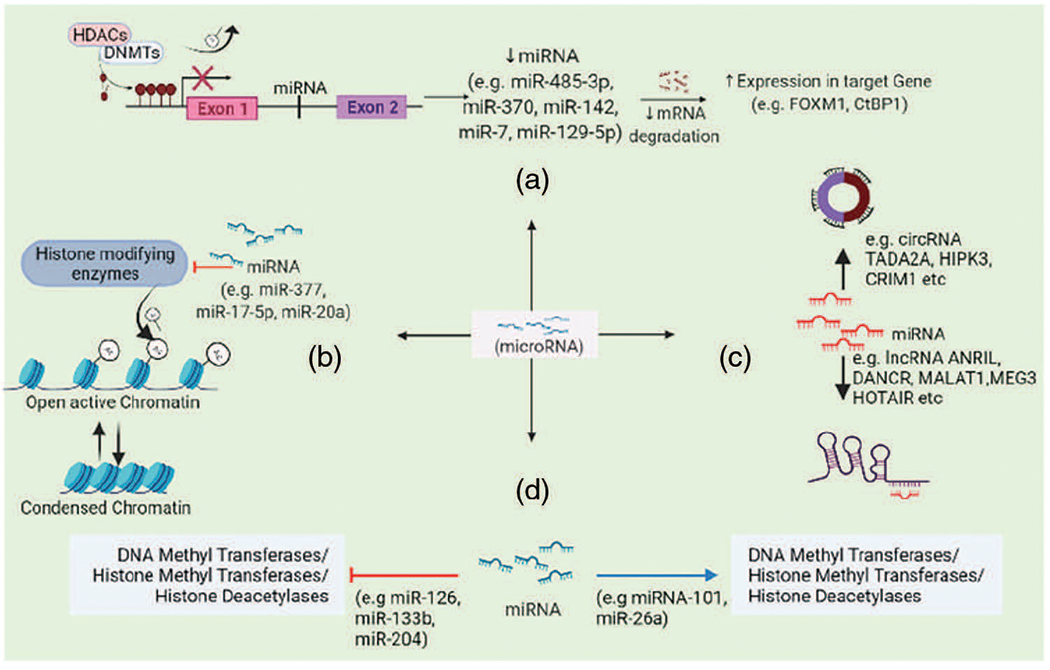
miRNAs affect gene expression by (a) regulating histone modifying enzymes activity and DNA methylation, (b) altering chromatin structure, (c) ceRNA networking mechanisms, (d) regulating onco- or tumor-suppressor genes expression via miRNA sponging.

An assessment of the clinical utility of miRNA sets and their association with methylation status (Hill et al., 2017) found that most prognostic miRNAs affecting gene expression via DNA methylation, cluster in ***14q32***-a region which was also previously reported to encode more than 40 miRNAs including imprinted genes important in osteogenic differentiation and inhibiting cancer (Thayanithy et al., 2012). This report suggests that miRNAs and modulation in methylation patterns may offer prognostic and therapeutic strategies in osteosarcoma treatment (Lietz et al., 2020). The hypothesis that miRNA may regulate gene expression epigenetically was reinforced by the relationship of certain miRNAs and DNA methyltransferases, for example-miR-485-3p, MiR-370, miR-142, miR-7, miR-129-5p (Ding et al., 2015; Du et al., 2018; Fabbri et al., 2007; Khraiwesh et al., 2010; Wu et al., 2010; Zhang and Peng, 2017; Zhang et al., 2017a; Zhang et al., 2019) (Fig. 3).

Few other miRNAs have been found targeting the histone modifying enzymes such as HDAC4 or Sirtuin-1 and thus inhibiting proliferation, migration, invasion and epithelial-mesenchymal transition of osteosarcoma cells, for example, miR-126, miR-133b, miR-204 (Liao et al., 2021; Shi et al., 2017; Tang et al., 2017; Ying et al., 2017). Tumor suppressor miR-449a and miR-449b were epigenetically repressed in the osteosarcoma cell line via H3K27me3 resulting in E2F1 deregulation. And their expression could be restored when targeted with a combination of small-molecule histone methylation inhibitor Deazaneplanocin A (DZNep) and HDAC inhibitor trichostatin-A (TSA) (Yang et al., 2009).

Another proposed mechanism of action is through forming heterochromatin (e.g., miR-377, miR-17-5p and miR-20a) (Gonzalez et al., 2008; Xia et al., 2019). miR-377, which is a well-recognized tumor suppressor miRNA in many cancers, has been found to target HAT1 (histone acetyltransferase 1) in osteosarcoma (Xia et al., 2019). And apparently upregulation of miR-377 or inhibition of HAT1 prevented osteosarcoma progression via inactivating Wnt pathway thereby providing a therapeutic alternative.

### Interaction with lncRNA and circRNA

Several miRNAs have been reported to interact with the lncRNAs as well as circRNAs to serve their oncogenic or tumor suppressive role in osteosarcoma (described in the previous sections). Significantly reduced serum miR-425-5p expression makes it a potential prognostic marker in osteosarcoma, and when overexpressed it decreases the expression of very well-known oncogenic lncRNA MALAT1 and TUG1 in addition to suppressed tumor growth *in-vivo* (Yang et al., 2019).

### Other signaling pathways governed by miRNAs

Several miRNAs have been reported to be involved in the Notch signaling pathway in the initiation and progression of osteosarcoma, of which some are tumor suppressor miRNAs such as miR-26a (targets Jagged1), miR-1296-5p (targets Notch2), miR-34 and miR-200 (targets Notch1) and some play oncogenic role for example, miR-10b-5p (targets NCOR2) (Lei et al., 2020; Xia et al., 2020). miR-154-5p acts as tumor suppressor in osteosarcoma and its upregulation inhibits the proliferation, migration and invasion *in vitro* as well as *in-vivo* tumor growth via the dysregulation in the pro-apoptotic proteins’ expression and the cell cycle regulators such as E2F5, Cyclin E1 and CDK2 (Tian et al., 2018). Another miRNA, miR-524 activates PI3K/AKT pathway and induces proliferation in osteosarcoma via directly inhibiting PTEN expression (Zhuang et al., 2018). Some miRNAs such as miR-598, miR-143, and miR-23a, also found to play a role in osteoblast differentiation in osteosarcoma progression (Grilli et al., 2015; Liu et al., 2017b).

## Short Non-Coding RNAs–piRNAs and siRNAs

piRNAs are approximately 26–30 NT long, consisting of more than 50,000 different species. Recent studies demonstrated possible involvement of aberrant piRNAs expression in tumorigenesis and all the hallmarks of cancer, and thereby suggested as a diagnostic and prognostic marker (e.g., piR-L-163, piR-823) (Wei et al., 2017; Wu et al., 2020). piRNAs been speculated to have a role in epigenetic regulation as they bind to Piwi proteins, a known epigenetic regulator functioning by binding to Polycomb group genes (Lin, 2007). piR-39980, which has been reported to have an oncogenic function in human osteosarcoma cells (Das et al., 2020), has been found to have strong anti-tumor activity in fibrosarcoma (early metastatic lethal tumor) by repressing RRM2 (Das et al., 2019). siRNAs can result in transcriptional gene silencing via DNA methylation and histone modifications in cells especially through interfering with EZH2 (Bayne and Allshire, 2005; Morris et al., 2004; Zhou et al., 2015).

## Non-Coding RNAs as Therapeutic Targets for Epigenetics-Driven Personalized Medicine

Epigenetic therapeutics in combination with the selective targeting of the ncRNAs might hold a great key for treating the cancers that are more chemoresistant and more prone to relapse after chemotherapy. In fact, studies focused on DNA methylation pattern for drug repurposing in osteosarcoma (Chaiyawat et al., 2020) identified a significant increase in DNMT1-dependent chemosensitivity toward Cisplatin therapies when treated with Decitabine (DNMT inhibitor).

The lncRNA HOTAIR (discussed in earlier sections) is an outstanding therapeutic target for anticancer therapies (Cantile et al., 2020; Li et al., 2017b). Recently, a computer-aided structure-based drug design method has been able to develop small molecule inhibitor of HOTAIR (e.g., AC1NOD4Q) which particularly interferes with the HOTAIR/EZH2 interaction and prevents tumor metastasis in breast cancer models (Ren et al., 2019a). Moreover, suppressing HOTAIR in combination with other epigenetic drugs (e.g., DZNep and AC1Q3QWB) showed a great promise in treatments for breast cancer and glioblastoma (Li et al., 2019; Sun et al., 2015). Its unique expression pattern in osteosarcoma, regulation of DNA methylation, exploiting chromatin remodelers, functioning via ceRNA network, and also promising outcomes in other cancers; all of these shows a great potential for HOTAIR to be an excellent candidate for epigenetic therapeutics in osteosarcoma (Cantile et al., 2020; Li et al., 2017b).

lncRNA NEAT1 (nuclear enriched abundant transcript 1) is another oncogenic transcript involved in the osteosarcoma metastasis and EMT regulation (Li and Cheng, 2018). NEAT1 induces epigenetic suppression of E-cadherin (CDH1) expression by mediating CDH1 promoter methylation via G9a methyltransferase. And when knocked down, it can significantly reduce G9a-DNMT1-Snail complex association in CDH1 promoter. Consequently, NEAT1 is yet another promising target in the treatment of metastatic osteosarcoma via epigenetic-derived therapeutics. Previously described lncRNA MEG3 was also suggested to be a potential therapeutic target in osteosarcoma due to its negative regulation of the well-known oncogene FOXM1 through sponging miR-361-5p (Shen et al., 2019). Other oncogenic lncRNAs such as AFAP-AS1 and MALAT1 (discussed previously) have also been proposed to be a therapeutic target due to their effects on osteosarcoma progression and metastasis (Fei et al., 2020).

Preclinical studies on osteosarcoma emphasized the potentials of different miRNAs as therapeutic targets (e.g., miR-146b-5p) (Jiang et al., 2019). Several miRNAs expression has also been associated with abnormal methylation that could be targeted with DNMT inhibitors to suppress osteosarcoma progression (e.g., miR-485-3p, miR-370, miR-142, miR-7, miR-129-5p) (Ding et al., 2015; Du et al., 2018; Long et al., 2015; Zhang and Peng, 2017; Zhang et al., 2019). Indeed, osteosarcoma cells when treated with DNMT inhibitor DAC, increased the levels of tumor suppressor microRNA miR-370. Not only that, DAC treatment also enhanced its inhibitory effect on FOXM1 by suppressing FOXM1-β-catenin interaction and inhibiting Wnt/β-Catenin signaling (Zhang et al., 2017b).

In a recent review, Lei et al. (2020) outlined the miRNA-based therapeutics in clinical trials as well as the miRNA mimics that are currently under development for targeting osteosarcoma both *in vitro* and *in vivo.* These includes nanoparticles, bioengineered prodrugs, and exosome-mediated delivery of ***miRNA mimics*** of miR-199a-3p, let-7a, miR-34a, miR-145, miR-143, and miR-101. A prodrug designed for miR-34a (tRNA/miR-34a) has shown substantial antitumor activity in preclinical canine model of osteosarcoma cell lines and *in vivo* xenograft model (Alegre et al, 2018), providing evidence for the potential of the miRNA-based therapies in the treatment of human osteosarcoma.

***miR-101*** is a well-known tumor suppressor miRNA in several cancers and in osteosarcoma, it was found functioning through repressing EZH2 expression to decrease metastasis (Zhang et al., 2014b). Recently, Zhang et al. (2020a) designed an exosomal delivery of miR-101 with EV derived from engineered AD-MSCs, and their study showed that miR-101 had the potential to inhibit metastatic osteosarcoma, possibly via regulation of EZH2 and BCL6.

## Conclusion

Identifying biomarkers that differentiate responders/survivors from non-responders remains enigmatic and could overcome the stagnate survival statistics that have persisted for the past 40 years. While biomarkers in the coding RNA and DNA have been unreliable, there is potential to investigate the ncRNA for consistent biomarkers. As discussed in this review, there are a number of potential biomarkers among the different classes of ncRNAs that not only serve as biomarkers to differentiate more aggressive osteosarcomas, but help to explain the biological processes that drive the hallmarks of cancer. By understanding the underpinnings of how ncRNAs drive transformation and progression, they can then become pharmacological targets to modulate cancer pathways and drive favorable outcomes for patients. The most advanced therapeutics in this field involve the complementary targeting of miRNAs that regulate numerous cell processes that regulate protumorigenic behavior. Currently several clinical trials are underway to investigate their therapeutic potential. However, as with most targeted therapies already applied to osteosarcoma, these therapies will also likely fail unless combined with other approaches. The heterogeneity and genomic instability that exists in the DNA coding regions is likely to complicate the interpretation of the ncRNAs, but the potential is there to discover something truly ground-breaking.

## Figures and Tables

**TABLE 1:** List of lncRNAs and their function in osteosarcoma

Oncogenic	Tumor suppressor	Targets (gene/protein/signaling pathway or miRNAs)
DANCR		EZH2/p21 p27; miR-335-5p and miR-1972/ROCK1
PVT1		MYC; ALKBH5-PVT1
ZFAS1		BMI1 and ZEB2
ANRIL		miR-125a-5p/STAT3
MALAT-1		EZH2/β-catenin and E-cadherin; PI3K/AKT; RhoA/ROCK; miR-509/Rac1; miR-142-3p/miR-129-5p/HMGB1; miR-140-5p/HDAC4
SNHG10		Wnt/β-catenin
AFAP1-AS1		RhoC/ROCK1/p38MAPK/Twist1; miR-497/IGF1R
HOTAIR		miR-126/CDKN2A/DNMT1; PRC2/HOXD
KCNQ1OT1		KCNQ1/DNMT1
ZEB1-AS1		ZEB1
FOXD2-AS1		EZH2/P21
HOXD-AS1		EZH2/P57
FOXP4-AS1		LSD1 and EZH2, LATS1
MIR100HG		EZH2, LATS1, LATS2, Hippo signaling pathway
B4GALT1-AS1		YAP
H19		Yap, Hedgehog signaling
AC011442.1		AMPK and hedgehog signaling
	MEG3	miR-361-5p/FOXM1, miR-664a; miR-127/ZEB1; Notch and TGF-β
	HIF1α-AS1	BRG1
	p21 (TRP53COR1)	miR-130b; PTEN
	CEBPA-AS1	Notch signaling pathway, NCOR2
	EPIC1	MEF2D
	NBAT1	miR-21/PTEN/PDCD4/TPM1/RECK
BLACAT1		STAT3
NEAT1		G9a-DNMT1-Snail (E-cadherin)

**TABLE 2:** List of circRNAs in osteosarcoma

Oncogenic	Tumor suppressor	Targets (gene/protein/signaling pathway or miRNAs)
circ_TADA2A		miR-203a-3p/CREB3
circ_CRIM1		miR-432-5p/HDAC4
	circ-CRIM1	miR-513
	circ_MYC	HDAC
circ_HIPK3		miR-637/HDAC4
	circ_HIPK3	
circ_0001658		miR-382-5p/YB-1
circ-LRP6		EZH2 and LSD1; APC and KLF2

**TABLE 3:** List of miRNAs, cluster, and families in osteosarcoma

Role in OS tumorigenesis	miRNAs, family, or clusters	Mechanism of action and targets (gene/protein/signaling pathway or lncRNA/circRNA)
Oncogenic	miR-524	PTEN99; PI3K/AKT
	miR-10b-5p	NCOR2
	miR-485-3p MiR-370 miR-142 miR-7 miR-129-5p	Interaction with DNA methyltransferases
Tumor suppressor	miR-126 miR-133b miR-204	HDAC4 or Sirtuin-1
	miR-449a miR-449b	E2F1; H3K27me3
	miR-377	Interaction with histone acetyltransferase 1
	miR-17-5p miR-20a	Heterochromatin formation
	miR-425-5p	Interaction with lncRNA
	miR-26a	Jagged1
	miR-1296-5p	Notch2
	miR-34	Notch1
	miR-200	Jagged1
	miR-154-5p	E2F5, Cyclin E1 and CDK2
	miR-598 miR-143 miR-23a	Osteoblast differentiation
	miR-370	FOXM1; Wnt/β-Catenin; DNA methylation
	miR-101	EZH2; DNA methylation

## Data Availability

Data sharing not applicable to this article as no datasets were generated or analyzed during the current study.
